# WSSS-CRAM: precise segmentation of histopathological images via class region activation mapping

**DOI:** 10.3389/fmicb.2024.1483052

**Published:** 2024-10-03

**Authors:** Ningning Pan, Xiangyue Mi, Hongzhuang Li, Xinting Ge, Xiaodan Sui, Yanyun Jiang

**Affiliations:** Shandong Normal University, Jinan, China

**Keywords:** histopathological image, precise semantic segmentation, weakly-supervised method, category-specific image activation maps, deep learning

## Abstract

**Introduction:**

Fast, accurate, and automatic analysis of histopathological images using digital image processing and deep learning technology is a necessary task. Conventional histopathological image analysis algorithms require the manual design of features, while deep learning methods can achieve fast prediction and accurate analysis, but rely on the drive of a large amount of labeled data.

**Methods:**

In this work, we introduce WSSS-CRAM a weakly-supervised semantic segmentation that can obtain detailed pixel-level labels from image-level annotated data. Specifically, we use a discriminative activation strategy to generate category-specific image activation maps via class labels. The category-specific activation maps are then post-processed using conditional random fields to obtain reliable regions that are directly used as ground-truth labels for the segmentation branch. Critically, the two steps of the pseudo-label acquisition and training segmentation model are integrated into an end-to-end model for joint training in this method.

**Results:**

Through quantitative evaluation and visualization results, we demonstrate that the framework can predict pixel-level labels from image-level labels, and also perform well when testing images without image-level annotations.

**Discussion:**

Future, we consider extending the algorithm to different pathological datasets and types of tissue images to validate its generalization capability.

## 1 Introduction

Cancer is a leading cause of death worldwide, with increasing incidence and mortality rates, and high treatment costs that impose a heavy burden on families and society (Sung et al., 2021; Ferlay et al., 2021). Histopathological slides are the gold standard for cancer diagnosis, providing not only basic information on tumor grading and subtype classification but also a wealth of information about the tumor microenvironment (TME). This not only plays a crucial role in explaining tumor development and metastasis but also in influencing the treatment outcomes and prognosis of cancer patients. Recent studies have found that the spatial organization of different tissues and cells is highly correlated with tumor progression, and TME features can reveal gene expression in biological pathways (Wang et al., 2020). Therefore, there is an urgent need for detailed segmentation of different tissues for further clinical research.

Clinically, histopathological slides are visually inspected by pathologists and evaluated semi-quantitatively, and the diagnostic results are reflected in the pathology report. Quantitative assessment for research purposes requires manual annotation by pathologists. However, the reproducibility and consistency of manual segmentation have been questioned due to inter-observer annotation differences and inter-observer variability (Wang et al., 2020). Due to the specific data storage format and large size of histopathological slides, specific tools need to be used for viewing and labeling, such as QuPath (Bankhead et al., 2017), which makes data annotation work difficult. In addition, manual annotation is very time-consuming and labor-intensive, requiring several days for detailed segmentation of each histopathological slide. Therefore, public research on histopathological image segmentation is usually limited to partial areas of pathological slides, or uses classification methods to achieve segmentation-like effects on whole-slice histopathological images (Lu et al., 2021; Yan et al., 2022; Pan et al., 2023), with very few studies focusing on tissue segmentation in whole-slide histopathological images (Cardenas et al., 2019; Amgad et al., 2022; Chan et al., 2019).

Therefore, it is imperative to develop fast and efficient methods for the rapid, accurate, and consistent delineation of target tissue areas. Semantic segmentation is a fundamental task in computer vision, and deep learning-based automatic segmentation frameworks have shown remarkable performance in medical image segmentation tasks (Hesamian et al., 2019; Xun et al., 2022), achieving outstanding results in various competitions. Popular models for this task include FCN (Long et al., 2015), U-Net (Ronneberger et al., 2015), V-Net (Milletari et al., 2016), nnU-Net (Isensee et al., 2021), among others. Furthermore, other hybrid models have also demonstrated excellent performance in medical image segmentation (Jin et al., 2021; Leube et al., 2023; He et al., 2023).

However, there are two major challenges in using deep-learning-based segmentation algorithms for histopathological image analysis tasks: (1) the performance of deep learning models heavily relies on the quality and quantity of annotated data, and histopathological image data is difficult to annotate, with pixel-level annotation being even more challenging; (2) tumors from different regions exhibit specificity, resulting in high costs for the transfer learning of trained networks.

Although high-quality pixel-level annotation data is scarce, coarse-grained or image-level annotation data is readily available. In fact, for the problem of analyzing histopathological images, there are publicly available datasets that can be downloaded and used for research, such as TCGA,^1^ which contains tumor and normal tissues from over 11,000 patients. The database provides image-level descriptions of entire tissue pathology slides and corresponding genomic sequencing results. To reduce the need for pixel-level annotated images during model training, researchers have proposed semi-supervised and weakly supervised learning models, which attempt to improve the model's performance by providing unlabeled or image-level annotated data and hoping to improve the model's generalization ability.

Drawing inspiration from weakly-supervised deep learning methods, we propose a weakly-supervised segmentation algorithm based on Class Region Activation Maps (CRAM) for tissue region segmentation in histopathological images. The framework utilizes image-level annotations to obtain Class Activation Maps (CAM) as pseudo-labels for semantic segmentation. The algorithm can be summarized into two main steps: (1) Obtain the CRAM: using a deep learning classification model, high-quality pixel-level pseudo-labels are generated based on image-level labels. (2) Train a segmentation model: the pixel-level pseudo-labels generated in step (1) are used as ground truth for model training. However, salient region activation can exhibit a higher response to a single class, while typically, multiple classes are present in one region of a pathological image. Therefore, this paper uses a Discriminative Activation (DA) layer to generate specific category masks for foreground and background, which serve as initial segmentation responses. To further increase the reliability of the pseudo-labels, this paper introduces a joint training method by merging the two steps into an end-to-end model. Furthermore, a joint loss function is adopted to optimize both branches and then improves the pseudo-labels' quality. Furthermore, an additional Conditional Random Field (CRF) operation is performed on the activation regions, which are modified into more reliable regions as pseudo-labels.

**Figure 1:**
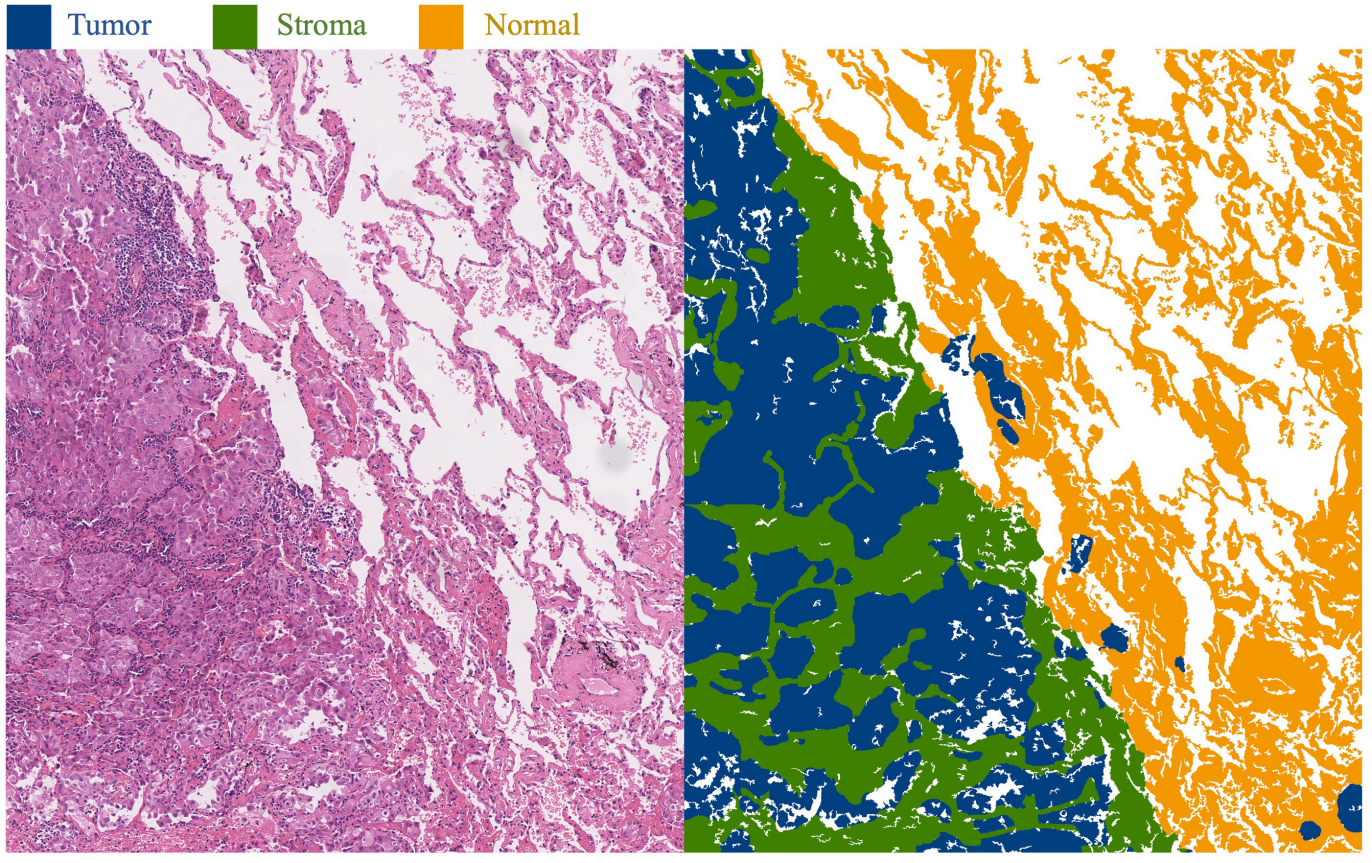
Histopathological images of lung adenocarcinoma tissue and their segmentation illustration. The blue area in the image represents the tumor region, the green area represents the stroma region, and the yellow area represents the normal region.

**Figure 2:**
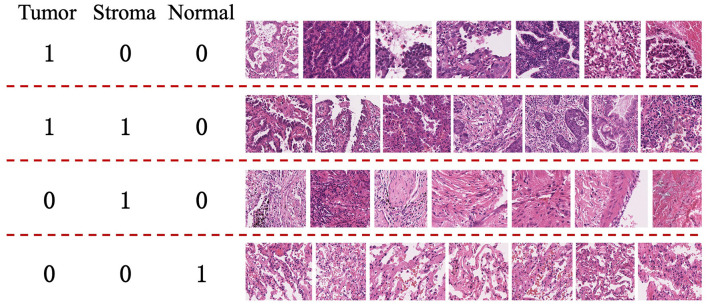
Examples from the training set of the WSSS4LUAD Challenge. 1 indicates the presence of the tissue in the image, while 0 indicates the absence of the tissue in the image. **Top row**: Tumor region; **Second row**: Tumor and stroma region; **Third row**: Stroma region; **Fourth row**: Normal region.

This approach primarily focuses on whole-slide images (WSI) of lung adenocarcinoma stained with H&E. The research dataset is sourced from the WSSS4LUAD^2^ challenge dataset, with the goal of achieving pixel-level segmentation for normal tissue, tumor epithelium, and tumor-associated stroma within the histopathological sections. Figure 1 presents image patches extracted from whole-slide pathology images of lung adenocarcinoma, scanned at a resolution of 0.2517μ*m*/*pixel* and 40 × magnification. Corresponding segmentation labels for the three prevalent tissue types are also provided. As depicted, these three tissue types may simultaneously appear within a single image patch, particularly tumor epithelium and tumor-associated stroma, since tumor cells often adhere to the stroma. Thus, tumors and stroma frequently coexist in the same image patch. Figure 2 displays examples from the training dataset, where each image patch is annotated with image-level labels indicating the presence of tumor, stroma, and normal tissue. The training dataset encompasses a total of 10,091 image patches. A comprehensive description of the dataset is presented in Section 4.1 of this paper.

Our main contributions are illustrated as follows: (1) Proposing a WSSS-CRAM that improves the traditional CAM method by activating corresponding regions for each class in the image, effectively utilizing the supervisory information of image-level labels. (2) Integrating the steps of obtaining pseudo-labels and training the segmentation model into an end-to-end model for joint training. (3) Performing additional post-processing on the activation regions, using a CRF operation to modify the activation regions into more reliable pseudo-label regions.

## 2 Related work

This paper centers on the main research subject of semantically segmenting tissue in lung adenocarcinoma. The pertinent techniques predominantly center on semi-supervised segmentation methods based on CAM. Therefore, before delving into the specifics of the methods, we initially introduce the task of region segmentation in histopathological images of tissues. Following that, we offer a concise analysis of pertinent research concerning semi-supervised segmentation methods.

### 2.1 Histopathological image segmentation

Since the emergence of whole-slide pathology scanning techniques, the utility of whole-slide tissue pathology imaging has been confirmed across various applications within the realm of pathology. Digitized tissue pathology images have facilitated tasks including remote expert consultations, prognostic analysis, and tumor biomarker assessment (Kumar et al., 2020). As scanning technologies and computational capacities have advanced, significant strides have also been made in the domain of tissue pathology image segmentation. Early approaches entailed manual feature extraction, employing models such as support vector machines and Bayesian models for the segmentation of tissue pathology images. For example, Hiary et al. (2013) employed a Bayesian model to automatically segment stromal tissue in breast tissue pathology images, leveraging color and texture attributes. With the advancement of deep learning techniques, the remarkable performance exhibited by deep learning in image segmentation has prompted its application in the segmentation of tissue pathology images. Among these techniques, FCN and U-Net have emerged as the most frequently employed foundational architectures. For instance, Chen et al. (2017a) introduced the utilization of a Deep Contour-Aware Network (DCAN) for the segmentation of colonic glands. This model incorporated auxiliary supervision mechanisms to tackle the challenge of gradient vanishing during training (Chen et al., 2017a). This approach secured the first rank in the 2015 MICCAI Gland Segmentation Challenge and the 2015 MICCAI Nuclei Segmentation Challenge. Oskal et al. (2019) employed a U-Net-based architecture to achieve a positive predictive value of 0.89 ± 0.16 and sensitivity of 0.92 ± 0.1 in epidermal or non-epidermal pixel classification tasks. In recent years, semi-supervised methods have also gradually been employed in tissue pathology image segmentation tasks to address the issue of limited annotated data (Jin et al., 2022).

Moreover, in recent years, various international competitions have introduced challenges related to the analysis of tissue pathology regions. For instance, the Digestive-System Pathological Detection and Segmentation Challenge (DigestPath 2019) held within MICCAI 2019 (Da et al., 2022; Li et al., 2019) was centered around automating the segmentation of benign and malignant regions within complete tissues. The Multi-organ Nuclei Segmentation and Classification Challenge (MoNuSAC) (Verma et al., 2021) in ISBI 2020 encompassed the identification and segmentation of multiple cell types across four organs. Additionally, the AGGC 2022 (Automated Gleason Grading Challenge) within MICCAI 2022 addressed the automatic segmentation of five tissue types in prostate cancer whole-slide pathology images.

### 2.2 Weakly-supervised semantic segmentation utilizing CAM

Instance segmentation, one of the most challenging problems in computer vision, has undergone extensive research (He et al., 2017; Arnab and Torr, 2017; Liu et al., 2018). However, many of these studies necessitate manual annotation of instance masks to provide strong supervision, thereby constraining their utility on datasets with sparsely annotated structures. Semi-supervised and weakly supervised instance segmentation strategies strive to transcend this constraint. In scenarios involving solely image-level categories, synthetic labels extracted from class response maps are harnessed to train networks for paired semantic segmentation (Ahn and Kwak, 2018). Employing a classification model to derive CAM stands as a standardized process for generating pseudo masks in the realm of Weakly Supervised Semantic Segmentation (WSSS).

#### 2.2.1 Class activation maps

The Vanilla CAM approach initially scales the feature map using fully connected weights learned for each individual class. Subsequently, seed masks are generated through channel averaging, spatial normalization, and thresholding (Zhou et al., 2016). The GAIN model applies CAM to the original image for mask generation, minimizing model prediction scores to capture features beyond the prior step's activation map in successive training rounds. This gradually refines the activated regions, ensuring complete coverage of the target area (Li et al., 2018). Recently emerged erase-based approaches also embrace similar principles (Zhang et al., 2018; Kweon et al., 2021). The distinction lies in their direct erasure of seed regions in CAM, followed by inputting the erased image into the model to generate the next round's CAM, expected to capture new regions. Moreover, certain schemes have been proposed to optimize CAM. For instance, in Qin et al. (2022), Activation Modulation and Recalibration Scheme (AMR) employs channel/spatial attention mechanisms for fine-tuning activation area calibration, thereby achieving adaptive modulation for segmentation-oriented activation responses. The ReCAM strategy reactivates CAM activation regions using Softmax Cross-Entropy Loss (SCL), resulting in ReCAM with Binary Cross-Entropy (BCE) constraints (Chen et al., 2022). Embedded Discriminative Attention Mechanism (EDAM) is a recent endeavor that employs CAM-based perturbations to optimize an additional classifier. It employs an extra DA layer to generate class-specific masks (Wu et al., 2021).

#### 2.2.2 Generation of pseudo-labels

The seed masks generated from CAM or its variations can undergo refinement steps to enhance the quality of pseudo-labels, employing both non-learning-based and learning-based methods. SEC introduced the principles of Seed, Expand, and Constrain for refining CAM, which have been widely adopted by subsequent works (Kolesnikov and Lampert, 2016). Among these, CRF is an earlier post-processing method that is user-friendly, independent of features extracted by the trained model, and relies solely on the original image features. DSRG, inspired by Seeded Region Growing (SRG), employs CAM as seeds to expand regions of interest (Huang et al., 2018). This approach integrates the SRG process into the deep segmentation network, deviating from the previous strategy of training segmentation models using pseudo-labels generated through SRG.

Learning-based methods introduce additional network modules. For example, AffinityNet employs a deep neural network to predict semantic affinities between adjacent image coordinates, achieving semantic propagation through random walks (Ahn and Kwak, 2018). IRNet estimates rough regions of individual instances and detects boundaries between different object classes. It focuses on pixel relations on the graph and computes affinities based on these relations (Ahn et al., 2019). Furthermore, incorporating confidence regions from saliency maps into CAM for pseudo-label refinement has become a common practice in recent methodologies (Chen et al., 2022; Wu et al., 2021). Approaches like OOA (Jiang et al., 2019) and CONTA (Zhang et al., 2020b) integrate CAM inferences generated through multiple training iterations, directing attention accumulation toward various parts of objects.

## 3 Methodology

In this section, the main focus is on introducing the CRAM algorithm framework. We provide a comprehensive explanation of the CNN-based pseudo-label acquisition module, the target semantic segmentation module, and the employed loss functions in the algorithm.

### 3.1 Framework

**Figure 3:**
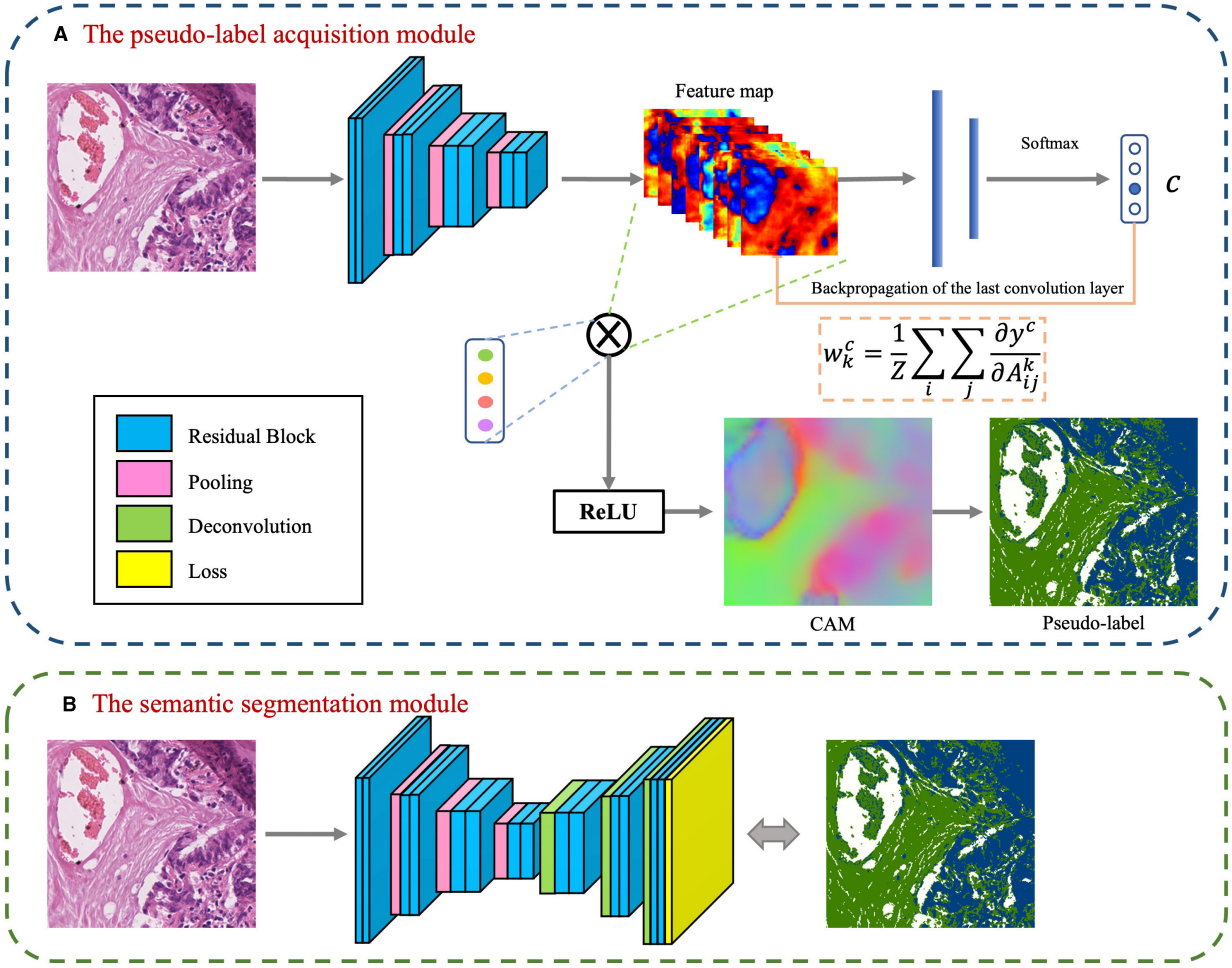
Basic conceptual diagram of CAM-based semi-supervised segmentation algorithm, which contains two main modules: **(A)** The pseudo-label acquisition module; **(B)** The semantic segmentation module.

The foundational model for the CAM-based semi-supervised segmentation algorithm used in this paper is divided into two distinct steps: pseudo-label acquisition and independent segmentation model training modules, as depicted in Figure 3. The pseudo-label acquisition module utilizes a standard image classification network supervised by image-level labels. By accentuating response areas of image-level labels through CAM, it generates pixel-level masks corresponding to each image, serving as pseudo-labels for the semantic segmentation module. The semantic segmentation module can be any end-to-end segmentation network, using the pixel-level pseudo-labels generated by the pseudo-label acquisition module as actual labels for training the model. During inference, segmentation predictions can be achieved solely by utilizing the semantic segmentation module.

**Figure 4:**
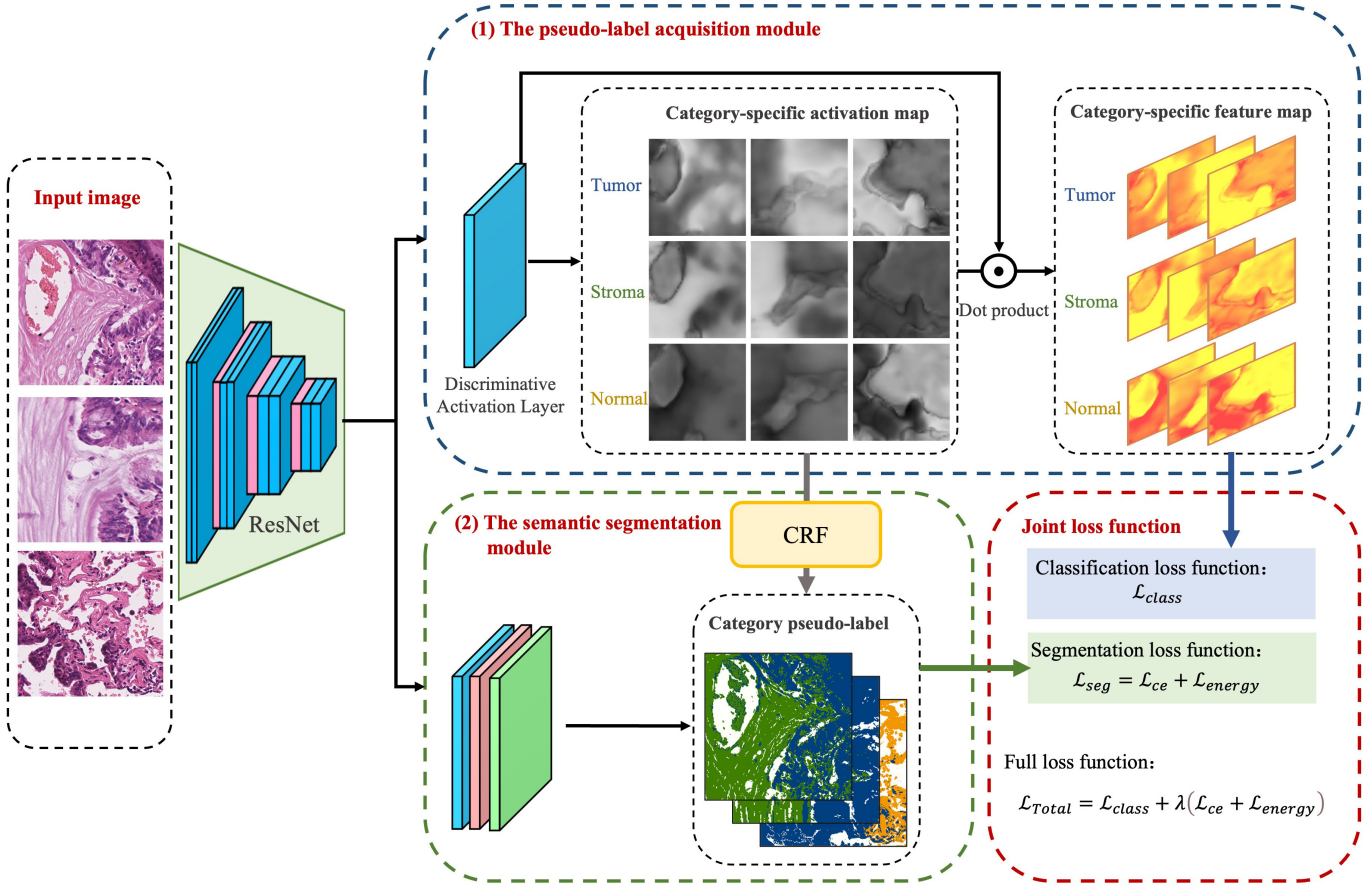
Weakly-supervised segmentation algorithm based on category activation regions.

The model presented in this paper is based on the algorithm outlined in Figure 3 and is divided into two primary modules: the pseudo-label acquisition module and the semantic segmentation module. Differing from the majority of previous methodologies that adopt independent two-step procedures, this paper amalgamates pseudo-label acquisition and semantic segmentation into a cohesive end-to-end model for joint training. As illustrated in Figure 4, following feature extraction by a backbone network, the image is directed to both the pseudo-label acquisition module and the semantic segmentation module. The integrated model is subject to joint training via a full loss function.

The pseudo-label acquisition module: Within this module, the model incorporates a DA layer to extract category-specific activation regions. Unlike CAM, which employs a single activation map for classification, the DA layer generates category-specific activation maps for each category. These category-specific activation maps are fused with the original feature layer to derive category-specific feature maps. The self-supervised layer explores collaborative information within and across images in a batch. Ultimately, classification predictions are made based on the collaborative information corresponding to each image. Given that all images in the training set are associated with image-level labels, a binary cross-entropy (BCE) loss function is employed independently for each category.

The semantic segmentation module: This module initially refines the feature maps extracted from the backbone network through a series of convolutional layers. Subsequently, an independent CRF is employed to enhance the category-specific activation maps obtained from the pseudo-label acquisition module. This refinement process helps eliminate mislabeled pixels, resulting in comparatively reliable pseudo-labels. The target semantic segmentation module applies cross-entropy loss and energy loss to the confident and non-confident regions of the pseudo-labels, respectively.

Joint loss function: The loss function is used to supervise the optimization of parameters within the model. In the presented algorithm, the classification and segmentation models are integrated into an end-to-end framework for joint training. As a result, the overall loss function comprises a binary cross-entropy loss for classification, as well as cross-entropy loss and energy loss for segmentation.

### 3.2 Pseudo-label acquisition based on CNN classification model

#### 3.2.1 Discriminative activation layer

For a given batch of data *X* = {(_*x*_*n*_, *l*_*n*_)}*N*_, where *N* represents the number of mini-batches, *x*_*n*_ represents the *n*-th image in this batch, and *l*_*n*_ represents the corresponding class label. It should be noted that *l*_*n*_ is represented as {0, 1}^*K*^, indicating image-level labels corresponding to *K* categories. Backbone network extracts the feature map $F_{n}\in {\text{R}}^{C\times H\times W}$ corresponding to image *l*_*n*_, where *C* represents the number of channels in the feature map, and *H* and *W* represent the height and width of the feature map. Connect the DA layer to generate activation maps $M_{n}\in {\text{R}}^{\left(K+1\right)\times H\times W}$ corresponding to *K* target categories. To explicitly represent the background region, in addition to generating activation maps for each category, the DA layer also generates activation maps corresponding to the background.

Applying L2-norm regularization to the activation map *M*_*n*_ can generate pixel-level probabilities for the corresponding class or background:


(1) $$\begin{array}{l} {\hat{M}}_{n}\left(i,j\right)=L2-\mathrm{norm}\left(\left|M_{n}\left(i,j\right)\right|\right). \end{array}$$

After the L2-norm regularization operation, ${\hat{M}}_{n}\left(i,j\right)$ represents the pixel-level class probability distribution at position (*i, j*), and ${\hat{M}}_{n}^{k}\left(i,j\right)$ represents the probability corresponding to class *k* at position (*i, j*). Through the above operations, activation maps corresponding to each category in the image are obtained.

#### 3.2.2 Self-supervised layer

Combining the feature map $F_{n}\in {\text{R}}^{C\times H\times W}$ corresponding to image *l*_*n*_ with the activation map ${\hat{M}}_{n}^{k}\left(i,j\right)$ corresponding to *K* target categories, generates feature maps for each class:


(2) $$\begin{array}{l} F_{n}^{k}=F_{n}\cdot {\hat{M}}_{n}^{k}, \end{array}$$

where $F_{n}^{k}$ is the feature map corresponding to category *k* in the image *l*_*n*_.

For a batch of *B* images, the corresponding feature maps are represented as $F^{k}=\left[F_{1}^{k},F_{2}^{k},\ldots F_{B}^{k}\right]\in {\text{R}}^{B\times C\times H\times W}$. After a 1 × 1 convolution, the feature maps are transformed into activation features ${\hat{F}}^{k}\in {\text{R}}^{1\times \left(B\times C\times H\right)\times d}$ corresponding to each category. The combination of activation maps with the initial feature maps is used to explore collaborative information specific to category activation maps. The self-supervised layer simultaneously considers feature attention within and between images in a batch, making the exploration of collaborative information more effective. The model generates category-specific feature maps for each category, using global average pooling and employing a specific classifier for label prediction of the given category. Since in histopathological images, one image often corresponds to multiple image categories, to make the activation regions corresponding to categories more effective, this paper transforms the multi-class problem into multiple binary classification problems.

The purpose of the self-supervised layer is to highlight similar regions in the activation maps corresponding to images in a batch through self-attention mechanisms, to obtain better activation maps for each category.

#### 3.2.3 Classification loss function

The category-specific features output by the self-supervised layer are mapped to categories through a fully connected layer, with image-level labels corresponding to the image as supervision. The classification loss function is represented as:


(3) $$\begin{array}{l} L_{class}=\frac{1}{B\times K}\overset{B}{\underset{n=1}{\sum }}\overset{K}{\underset{k=1}{\sum }}L_{BCE}\left(Linear\left(GAP\left(A_{n}^{k}\right)\right),l_{n}^{k}\right), \end{array}$$

where


(4) $$\begin{array}{l} \left[A_{1}^{k},A_{2}^{k},\ldots ,A_{B}^{k}\right]=\mathrm{SelfAttention}\left({\hat{F}}^{k}\right), \end{array}$$

where $A_{n}^{k}$ is the activation map corresponding to input image *x*_*n*_ after the self-supervised layer for the *k*-th category, $l_{n}^{k}\in \left[0,1\right]$ represents the true label of input image *x*_*n*_ corresponding to the *k*-th category. Since the input to the self-supervised layer is a combination of category-specific activation maps and initial feature maps, the loss function of the self-supervised layer will, through backpropagation, affect the distinguishing activation layers of all foreground categories, thereby influencing the parameter training of the backbone network.

### 3.3 Target semantic segmentation model

#### 3.3.1 Reliable semantic segmentation labels

From the pseudo-label acquisition module, activation maps corresponding to each category can be obtained, which highlight the regions where each category plays a role in classification. In this activation map, select the high-confidence foreground and background regions as reliable regions, and the remaining regions as unreliable regions. High-confidence maps are represented as:


(5) $$p^{r}(i,j)=\{\begin{array}{cc} {\hat{M}}^{k}(i,j), & \text{ if }{\hat{M}}^{k}(i,j)<\alpha \text{ or }{\hat{M}}^{k}(i,j)>\beta \\ 255, & \text{ else } \end{array}$$

where α and β represent pre-established thresholds. When the threshold falls below α, it signifies the region as a dependable background area; conversely, when the threshold surpasses β, the region is retained as a foreground area.

We employ CRF for post-processing the activation maps, removing incorrectly labeled pixels, and enhancing the probability maps associated with each category:


(6) $$\begin{array}{l} p^{crf}=CRF\left(x,\hat{M}\right). \end{array}$$

Taking into account the constraints imposed by CRF on the activation maps, the ultimate pixel-level pseudo-labels are as follows:


(7) $$p^{pseudo}(i,j)=\{\begin{array}{cc} p^{r}(i,j), & \text{ if }p^{r}(i,j)=p^{crf}(i,j)\\ 255, & else. \end{array}$$

If *p*^*r*^(*i, j*) = *p*^*crf*^(*i, j*), signifying alignment between the high-confidence map and the CRF activation map, we retain this region as the confident pseudo-label area, with the rest designated as non-confident pseudo-label areas.

#### 3.3.2 Segmentation loss function

The pseudo-labels generated by the model serve as the ground truth labels for training the semantic segmentation module, encompassing both areas with high-confidence pseudo-labels and areas with low-confidence pseudo-labels.

In the case of confident pseudo-label regions, the model utilizes the standard cross-entropy loss function, denoted as:


(8) $$\begin{array}{l} L_{ce}=-\underset{\left(i,j\right)\in \varphi }{\sum }B\left(i,j\right)\log\left(P_{net}^{k}\left(i,j\right)\right), \end{array}$$

where *B*(*i, j*) is a binary label indicating whether the label belongs to class *k*. φ represents the confident pseudo-label region, i.e., when *p*^*pseudo*^(*i, j*)≠255. $P_{net}^{k}\left(i,j\right)$ represents the prediction of the segmentation model.

The model utilizes the dense energy loss function (Zhang et al., 2020a), applied to both confident and non-confident regions, and it is represented as:


(9) $$\begin{array}{l} L_{energy}=\overset{H,W}{\underset{i=0,j=0}{\sum }}\overset{H,W}{\underset{\begin{array}{c} a=0,b=0\\ \left(i,j\right)\neq \left(a,b\right) \end{array}}{\sum }}S\left(i,j\right)E\left(\left(i,j\right),\left(a,b\right)\right), \end{array}$$

where *S*(*i, j*) represents a soft filter. For regions with confident pseudo-labels, soft filter weights are determined based on the model's predicted class probabilities. In contrast, for regions with non-confident pseudo-labels, a dense energy loss is employed. Cross-entropy loss functions are designed for hard labels, while the pseudo-labels used in this study are not guaranteed to be 100% accurate. Therefore, applying the cross-entropy loss directly to confident regions could introduce errors during model training. The dense energy loss function, using a soft labeling strategy for confident regions, allows for further refinement of the confident regions generated in the preceding step. *S*(*i, j*) is defined as:


(10) $$S(i,j)=\{\begin{array}{cc} 1-\max_{k\in K}\left(P_{net}^{k}(i,j)\right), & (i,j)\in \varphi \\ 1, & \text{ else } \end{array}$$

Here, *E*((*i, j*), (*a, b*)) represents the energy formula that characterizes the relationship between pixel (*i, j*) and pixel (*a, b*):


(11) $$\begin{array}{l} E\left(\left(i,j\right),\left(a,b\right)\right)=\underset{\begin{array}{c} k_{1},k_{2}\in K\\ k_{1}\neq k_{2} \end{array}}{\sum }G\left(\left(i,j\right),\left(a,b\right)\right)P_{net}^{k_{1}}\left(i,j\right)P_{net}^{k_{2}}\left(a,b\right), \end{array}$$

where *G*((*i, j*), (*a, b*)) is a Gaussian filter.

The total loss function associated with the target semantic segmentation network comprises both cross-entropy loss and energy loss:


(12) $$\begin{array}{l} L_{seg}=L_{ce}+L_{energy}. \end{array}$$

### 3.4 Joint loss function

The approach presented in this paper integrates classification and segmentation models into an end-to-end framework. The overall loss function comprises the $L_{class}$ loss function from the pseudo-label acquisition module and the $L_{seg}$ loss function from the semantic segmentation network. The combined loss function is shown below:


(13) $$\begin{array}{l} L_{Total}=L_{class}+\lambda L_{seg}=L_{class}+\lambda \left(L_{ce}+L_{energy}\right), \end{array}$$

where λ is a weighting coefficient that controls the balance between the pseudo-label acquisition module and the target segmentation module.

### 3.5 Independent semantic segmentation model

After weakly-supervised training, the combination of the backbone network and the target semantic segmentation network can serve as an independent inference module for generating semantic segmentation results during the testing phase. Alternatively, the model proposed in this paper can be used as a whole for pseudo-label acquisition. During the training phase, the segmentation model outputs optimized region segmentation results, which are used as artificial pseudo-labels for an independent semantic segmentation model.

Define an independent semantic segmentation module: This semantic segmentation module is designed as a standalone component, utilizing pseudo-labels obtained from the previous step's image classification model as training labels for the training model. During the final inference phase, running inference is as simple as using this trained model. The standalone segmentation model can employ any end-to-end semantic segmentation model as its backbone network, such as FCN (Long et al., 2015), U-Net (Ronneberger et al., 2015), DeepLab v3 (Chen et al., 2017b), and so on. In this paper, we draw inspiration from previous research in weakly-supervised segmentation, where the semantic segmentation module combines the ResNet model and the DeepLab v3 model. This network model consists of two parts: an Encoder based on the ResNet model and a Decoder based on the DeepLab v3.

## 4 Algorithm validation and evaluation

### 4.1 Datasets

The dataset used in this paper is publicly available data from the WSSS4LUAD challenge (Han et al., 2022a,b), which includes 67 H&E (Hematoxylin and eosin)-stained WSI (Whole Slide Images) from the Guangdong Provincial People's Hospital (GDPH) and 20 WSI images from the TCGA public dataset. These images have annotations for three common and meaningful tissue types: tumor epithelial tissue, stromal tissue, and normal tissue.

The training dataset in this dataset consists of 63 WSI (49 from GDPH and 14 from TCGA), from which 10,091 image patches were cropped and selected. The image size ranges from 150 × 150 to 300 × 300. Each image in the training set has image-level annotations in the form of a three-digit label [tumor, stroma, normal]. It includes 6,579 images of tumor tissue, 7,076 images of stromal tissue, and 1,832 images of normal tissue. The most common label is [1,1,0], indicating images containing both tumor and stroma, with a total of 5,393 images. This is followed by 1,832 images with the [0,0,1] label (indicating normal tissue), 1,680 images with the [0,1,0] label (indicating stromal tissue), and 1,181 images with the [1,0,0] label (indicating tumor tissue).

The validation set comprises 12 WSI (9 from GDPH and 3 from TCGA), from which 40 image patches are cropped. These include 9 large image patches ranging in size from 1,500 × 1,500 to 5,000 × 5,000 and 31 small image patches ranging in size from 200 × 200 to 500 × 500. The validation dataset has pixel-level labels and is used to validate the trained models.

The test set also consists of 12 WSI (9 from GDPH and 3 from TCGA), from which 80 image patches are cropped. These include 14 large image patches ranging in size from 1,500 × 1,500 to 5,000 × 5,000 and 66 small image patches ranging in size from 200 × 200 to 500 × 500. The test dataset has pixel-level labels and is used for the final model testing.

### 4.2 Experimental settings

This experiment was conducted in a PyTorch environment, utilizing NVIDIA CUDA (version 11.4) and cuDNN library (version 8.2.2). All experiments were performed on a computer running Ubuntu 20.04 LTS, using 4 NVIDIA Tesla A100 GPUs with 40GB of VRAM each. The model's backbone network was pre-trained on the ImageNet dataset and further fine-tuned on the target dataset used in this paper.

The model used an SGD optimizer with a batch size of 8, an initial learning rate of 0.001, weight decay set to 0.0002, and momentum set to 0.9. Two hyperparameters, α and β, were set to 0.3 and 0.9, respectively.

During both training and testing, a CRF operation was used to generate refined labels, with parameters following the default values as described in Huang et al. (2018). During training, the loss functions computed by the classification and segmentation modules were updated through backpropagation to update the backbone network. During testing, only the segmentation module was used to generate region segmentation corresponding to the images.

Considering the irregular sizes of image patches in this dataset, they were standardized through resizing before being fed into the model. During the training phase, the image dimensions were initially randomly increased to two to three times their original size. Subsequently, these enlarged images were uniformly cropped to a size of 513 × 513 pixels, serving as the input images for the model. In the testing phase, the image dimensions were enlarged to 2.5 times their original size, and the model made predictions and generated segmentation results based on the enlarged images. Due to limitations in GPU VRAM, particularly with extremely large pixel images, they were proactively cropped to a fixed size (ranging from 400 × 400 to 500 × 500 in this paper). The model's predicted results were then combined for visualization purposes.

### 4.3 Performance evaluation metrics

In the experiments, model evaluation is performed using the mean Intersection over Union (mIoU), which is expressed as follows:


(14) $$\begin{array}{l} mIoU=\frac{1}{k+1}\overset{k}{\underset{k=0}{\sum }}\frac{TP}{FN+FP+TP} \end{array}$$

where TP stands for true positives (correctly predicted positive instances), while FN and FP represent false negatives (positive instances incorrectly predicted as negative) and false positives (negative instances incorrectly predicted as positive), respectively. The variable *k* denotes the number of classes. In our experiments, the test dataset includes a background label. Therefore, when computing the final mIoU, the background region is excluded and not included in the calculation area.

### 4.4 Model analysis

#### 4.4.1 Comparison with state-of-the-art methods

Table 1 presents a comparison between our proposed method with the existing fully supervised baseline segmentation methods and the top three performers in the WSSS challenge, including ChunhuiLin, baseline0412, and Vison307, with the best result highlighted in bold. The fully supervised approach was trained using training data containing only one tissue category, with [1,0,0], [0,0,1], and [0,1,0] corresponding to 1,181, 1,832, and 1,680 images, respectively. Among the comparison weakly-supervised methods, including ChunhuiLin, baseline0412, and Vison307 are semi-supervised methods. Training details can be found in the paper (Han et al., 2022a). WSSS-CRAM1 entails training a model exclusively using image-level labels from the training set, without any reference to pixel-level labels from the validation set throughout the training process. Building upon jointly optimized pseudo-labels, WSSS-CRAM2 establishes a separate segmentation module to learn pixel-level pseudo-labels, the model is shown in Figure 3. In contrast, WSSS-CRAM3 incorporates pixel-level labels from the validation set as a supervisory condition when training a separate segmentation model with pseudo-labels. Notably, our proposed approach, when training a dedicated semantic segmentation module and incorporating pixel-level labels from the validation set into the model training, achieves results differing by a mere 0.0012 from the competition's top performance, indicating a remarkable quantitative proximity. This outcome may be attributed to the omission of weight consideration for pseudo-labels compared to the known labels from the validation set during the model training process.

**Table 1:** Comparison with the state-of-the-art methods.

**Model**		**mIoU**	**Tumor**	**Stroma**	**Normal**
Supervised	U-Net (Ronneberger et al., 2015)	0.5362	0.4158	0.7075	0.4854
	ResNet101 (He et al., 2016)	0.5992	0.5312	0.7323	0.5342
	DeepLab v3 (Chen et al., 2017b)	0.6222	0.5859	0.7318	0.5489
Weakly-supervised	ChunhuiLin	**0.8413**	0.8389	0.8919	0.7931
	baseline0412	0.8222	**0.8402**	0.8343	0.7921
	Vison307	0.8058	0.8165	0.8554	0.7456
	WSSS-CRAM1	0.7265	0.7074	0.8125	0.6597
	WSSS-CRAM2	0.7618	0.7493	0.8237	0.7125
	WSSS-CRAM3	0.8401	0.8293	**0.8923**	**0.7987**

Bold values indicates best result obtained for predictions.

#### 4.4.2 Ablation experiment

Table 2 presents the results of ablation experiments aimed at demonstrating the effectiveness of our method's design. To maintain control over the variables in these experiments, we focused solely on the acquisition of pseudo-labels. In this process, pseudo-labels obtained from the training dataset were combined with pixel-level annotated labels from the validation data to train separate semantic segmentation modules. It's worth noting that CAM, which serves as the foundational strategy for obtaining pixel-level labels from image-level labels, was included in all ablation models. As observed in the table, the joint optimization of segmentation and classification modules yields a significant improvement in segmentation performance. Furthermore, the strategy of DA layer and CRF also contributes to enhancing segmentation performance.

**Table 2:** Ablation experiments for each module in the network.

**Joint optimization**	**CAM**	**DA**	**CRF**	**mIoU**
	✓			0.6925
	✓	✓		0.7680
	✓	✓	✓	0.7912
✓	✓			0.7684
✓	✓	✓		0.8059
✓	✓	✓	✓	**0.8401**

Bold values indicates best result obtained for predictions.

### 4.5 Visualized results

#### 4.5.1 Visual presentation of results

**Figure 5:**
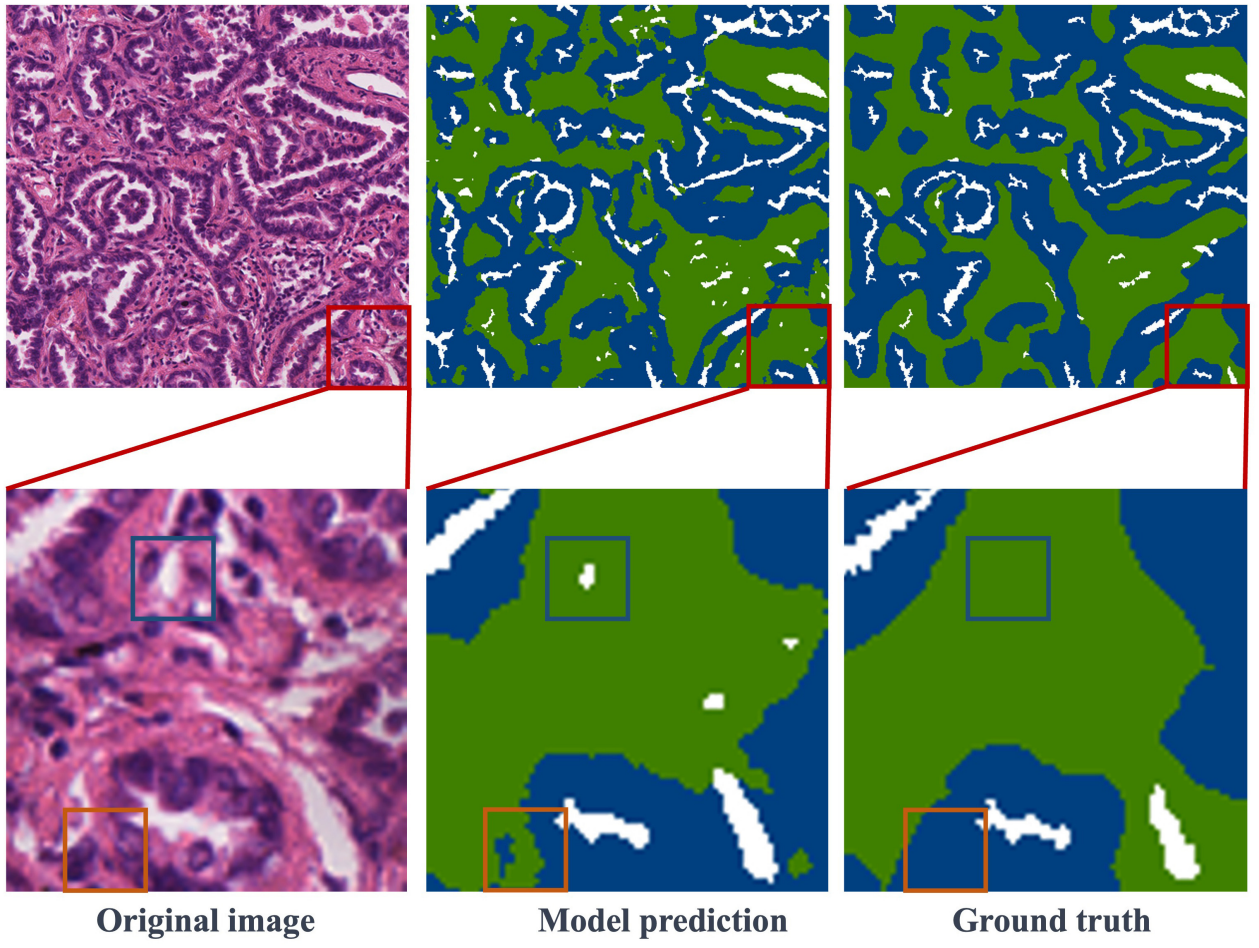
Visualization of segmentation results: the first column features the original images, the second column showcases the model's predictions, and the third column reveals the ground truth labels. Notably, red boxes highlight representative regions, which are further magnified in the second row.

Figure 5 presents the segmentation results obtained in the test dataset. The first column contains image blocks extracted from the overall histopathological image, the second column showcases the model's predictions, and the third column displays the ground truth labels. In the second row, specific details from the first-row images have been selectively magnified for closer inspection. The result images clearly demonstrate a close alignment between the model's predictions and the ground truth labels. The trained model exhibits the capability to accurately segment regions within histopathological images of lung adenocarcinoma. In the second row of enlarged images, regions, where the model's predictions deviate from the ground truth labels, are enclosed within blue and orange rectangles. Upon a closer examination of the original images, it becomes apparent that the region inside the blue rectangle corresponds to a blank area in the original image, whereas the region within the orange rectangle should indeed be labeled as stroma, consistent with the ground truth. While this result may differ from the manually annotated ground truth, it may offer a more precise representation of the intricate details in comparison to the human-labeled labels. This comparative analysis indicates that the model not only learns pixel-level annotations from image-level labels but also excels in accurately predicting tissue boundaries and intricate details.

**Figure 6:**
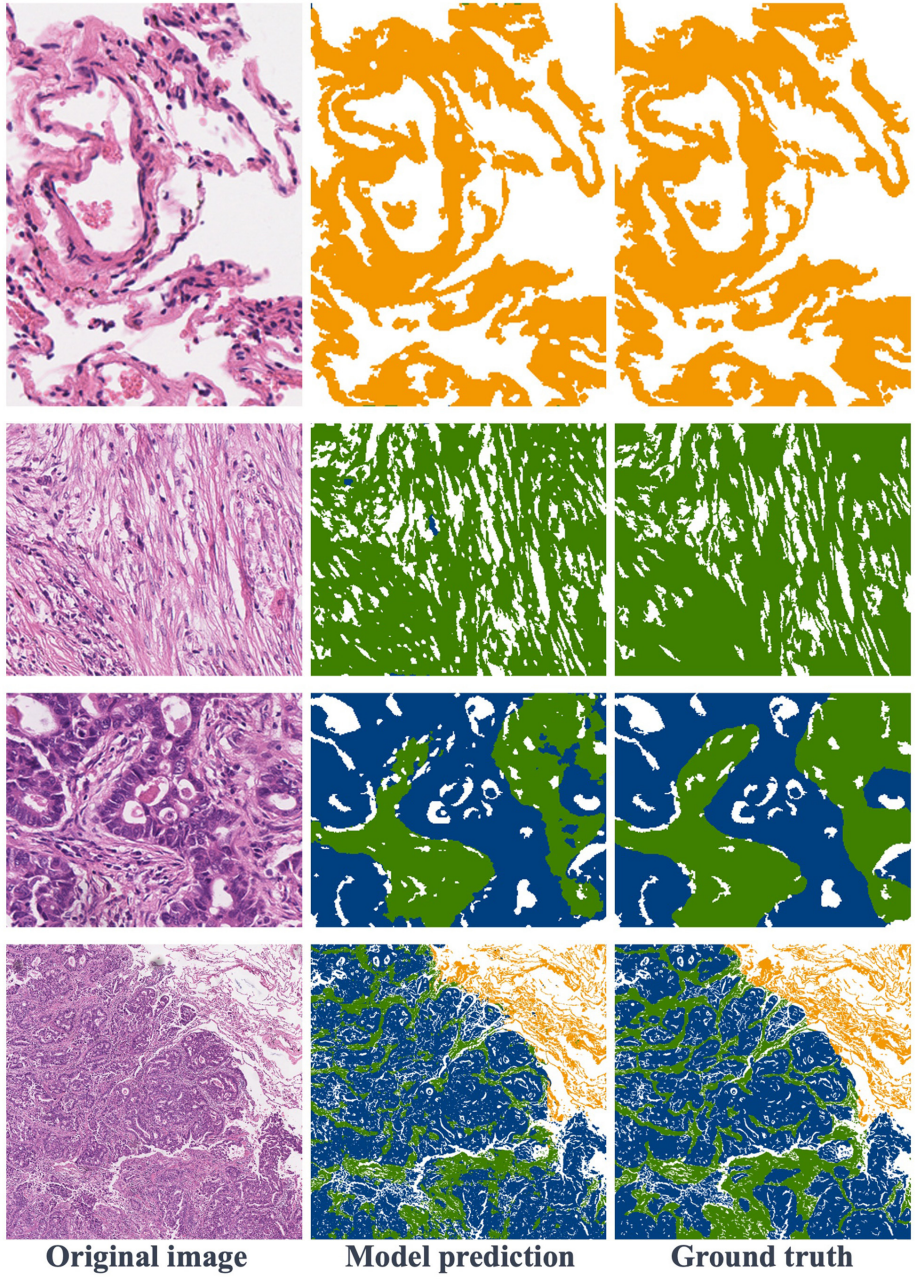
Visualization of segmentation results: the first row is normal regions, the second row is stroma regions, the third row includes stroma and tumor, and the fourth row features a large image containing normal, stroma, and tumor areas.

Figure 6 showcases various segmentation examples from the test dataset. In the first column, you'll find the original images, while the second column reveals the model's predictions, and the third column displays the ground truth labels. The result images clearly depict that the first and second rows represent image blocks from normal and stroma regions, respectively. In these cases, the model excels in delivering remarkably accurate predictions that closely align with the ground truth labels. Moving to the third row, we encounter images featuring the coexistence of tumors and stroma. Upon close examination, it becomes apparent that the model also produces relatively precise predictions, with minor boundary prediction errors occurring solely at the edges of the tumor and stroma regions. Finally, in the last row of Figure 6, this is a typical example of a large image block, encompassing tumor, stroma, and normal areas. The model's prediction results affirm that the proposed method consistently yields precise segmentation results, even for intricate histopathological images.

#### 4.5.2 Category-specific activation maps from discriminative activation layer

**Figure 7:**
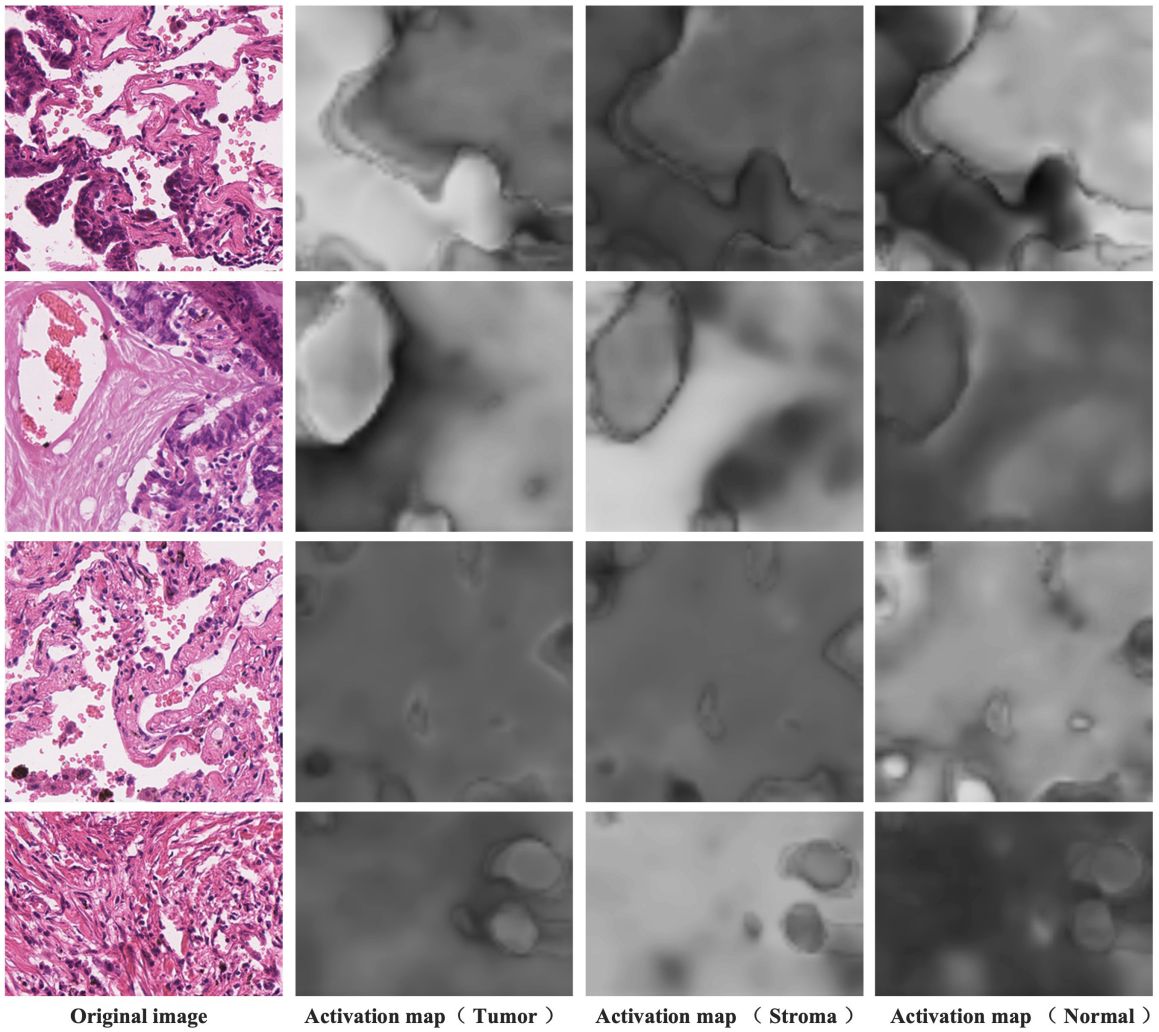
Category-specific activation maps corresponding to the tumor, stroma, and normal regions.

Figure 7 presents the activation maps generated by the model after differentiating the activation maps from the activation layer output. The data showcased here is sourced from the training dataset and, therefore, lacks corresponding pixel-level annotations. The four examples shown correspond to image-level labels [1,0,1], [1,1,0], [0,0,1], and [0,1,0], representing tumor and normal, tumor and stroma, normal, and stroma, respectively. In the first column, you can see the original images, while the second column displays the activation maps for tumor regions, the third column displays the activation maps for stroma regions, and the fourth column reveals the activation maps for normal regions. Higher brightness in the activation maps indicates a higher probability of the corresponding region belonging to that class. From these images, it's evident that distinguishing the activation layer enables the generation of activation regions corresponding to each class. Remarkably, even without the explicit use of pixel-level annotations during training to inform the model about specific regions as the tumor, stroma, or normal, weakly supervised learning using only image-level labels demonstrates the ability to produce pixel-level activations, showcasing a crucial feature of CAM.

#### 4.5.3 CRF refinement of pseudo-labels

**Figure 8:**
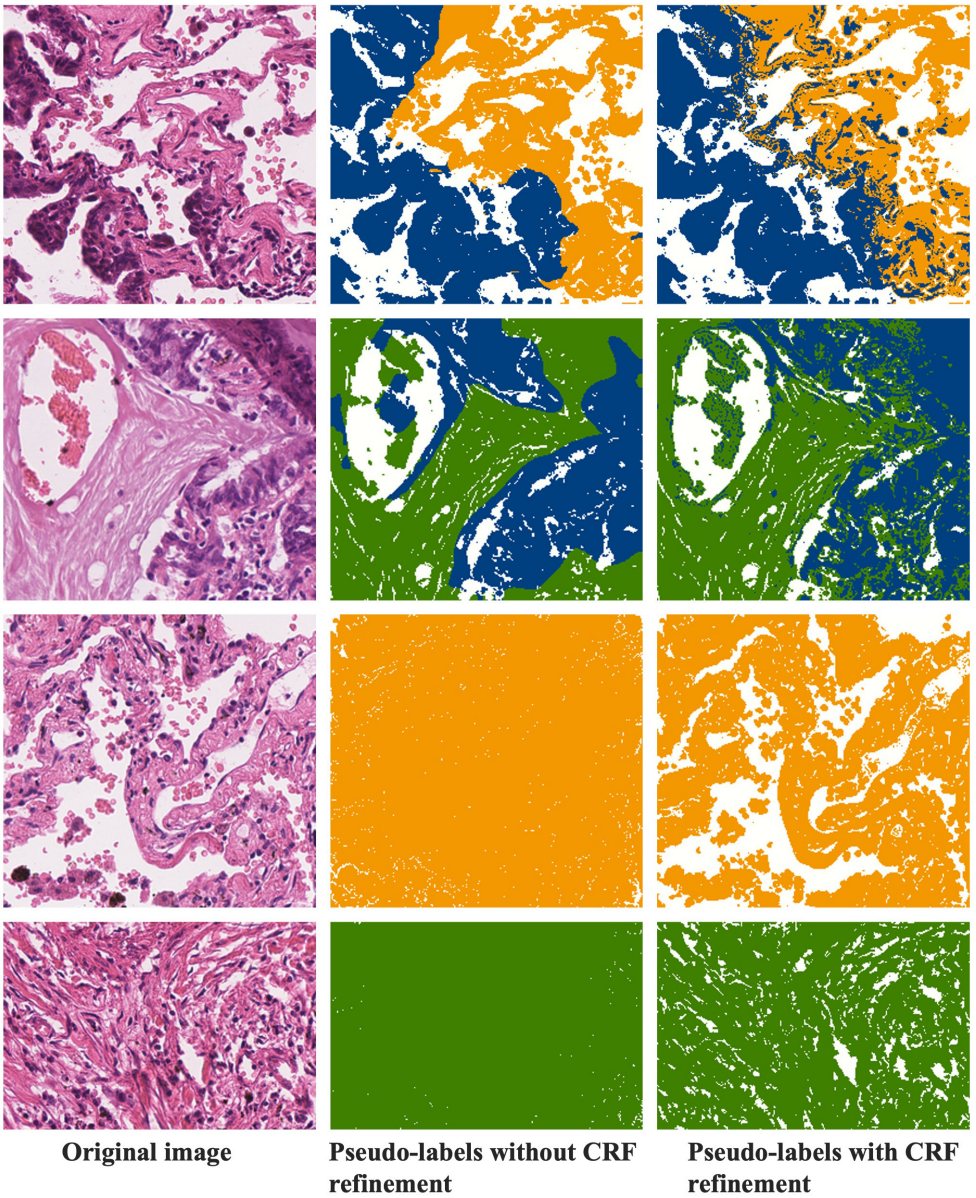
Comparison of pseudo-labels before and after CRF operations.

Figure 8 demonstrates the refinement of pseudo-labels through CRF operations. The first column showcases the original images, the second column displays the pseudo-labels before CRF refinement, and the third column reveals the pseudo-labels after CRF refinement. Let's compare the state of the labels before and after CRF operations based on these results. From the examples in the first row, it's evident that the pseudo-labels before CRF refinement exhibit distinct boundaries between tumor and normal regions but overlook individual tumor cells present in the finer details. CRF operations, guided by the original image, rectify these boundaries, resulting in a more precise demarcation between tumor and normal regions. In the second row of examples, it becomes apparent that CRF not only refines details but also corrects more extensive areas of segmentation error. The third and fourth rows represent normal and stromal tissues, and a comparison with Figures 4, 5 reveals that activation maps can emphasize specific classes without clearly defined activation boundaries for the image's boundary details. Consequently, in the pseudo-labels of the second column, only the categories are nearly discernible. After undergoing CRF operations, the distinctions between foreground and background become much clearer.

## 5 Conclusion

This paper proposes a novel weakly-supervised segmentation method based on class region activation mapping, effectively achieving the segmentation of tissue regions in lung adenocarcinoma pathological images. The paper incorporates distinguishing activation layers and self-supervised layers into the classification network to predict activation maps corresponding to each category in the image and explore inter-image collaborative information. Subsequently, pseudo-labels generated from the activation maps are used as training labels for the target semantic segmentation module. The fusion of the pseudo-label prediction module and the target segmentation module allows for better utilization of pixel-level segmentation of target regions with image-level labels. Experimental results on the test set of a publicly available lung adenocarcinoma dataset validate the performance of the weakly-supervised segmentation algorithm based on category-specific activation. Compared to traditional weakly-supervised semantic segmentation methods based on category activation maps, this algorithm exhibits a significant improvement in segmentation accuracy in the literature.

The algorithm has only been validated on a lung adenocarcinoma dataset. Although the algorithm performs well on the lung adenocarcinoma dataset, its generalization ability to other diseases or types of tissue images has not been verified. Therefore, the method's performance on other image datasets may not be as expected. Future, we consider extending the algorithm to different pathological datasets and types of tissue images to validate its generalization capability. Consider integrating pathological images with other types of medical imaging (e.g., CT, MRI) for multimodal analysis to enhance diagnostic accuracy and the applicability of the model.

## Data Availability

The original contributions presented in the study are included in the article/supplementary material, further inquiries can be directed to the corresponding author.

## Footnotes

^1^ https://www.cancer.gov/about-nci/organization/ccg/research/structural-genomics/tcga

^2^ https://wsss4luad.grand-challenge.org/
